# SARS-Cov-2 spike induces intestinal barrier dysfunction through the interaction between CEACAM5 and Galectin-9

**DOI:** 10.3389/fimmu.2024.1303356

**Published:** 2024-04-15

**Authors:** Yingshu Luo, Zhenling Zhang, Jiangnan Ren, Chunxu Dou, Jiancheng Wen, Yang Yang, Xiaofeng Li, Zhixiang Yan, Yanzhi Han

**Affiliations:** ^1^ Department of Gastroenterology, The Fifth Affiliated Hospital, Sun Yat-sen University, Zhuhai, Guangdong, China; ^2^ Guangdong Provincial Key Laboratory of Biomedical Imaging and Guangdong Provincial Engineering Research Center of Molecular Imaging, The Fifth Affiliated Hospital, Sun Yat-sen University, Zhuhai, Guangdong, China

**Keywords:** CEACAM5, Galectin-9, COVID-19, SARS-CoV-2 spike protein, intestinal barrier dysfunction, CD4+ T lymphocytes

## Abstract

**Background:**

Carcinoembryonic antigen-related cell adhesion molecule 5 (CEACAM5), as a typical tumor marker, has been found to exert immunomodulatory effects in many diseases. We previously reported the clinical and molecular evidences supporting that SARS-Cov-2 infected the gastrointestinal (GI) tract and found a reduction of CEACAM5 in COVID-19 patients’ feces which associated with gut dysbiosis. Yet the role of CEACAM5 in GI infection is ill-defined.

**Methods:**

Mice models were established through intraperitoneally injecting with recombinant viral spike-Fc to mimic the intestinal inflammation. We collected duodenum, jejunum, ileum and colon samples after 6h, 2 days, 4 days and 7 days of spike-Fc or control-Fc injection to perform proteomic analysis. Blood was collected from healthy donors and peripheral blood mononuclear cells (PBMC) were separated by density gradient centrifugation, then CD4+ T cells were isolated with magnetic beads and co-cultured with Caco-2 cells.

**Results:**

In addition to intestinal CEACAM5, the expression of tight junction and the percent of CD4+ T lymphocytes were significantly decreased in spike-Fc group compared to control (p < 0.05), accompanied with increased level of inflammatory factors. The KEGG analysis revealed differentially expressed proteins were mainly enriched in the coronavirus disease (COVID-19), tight junction, focal adhesion, adherens junction and PI3K-Akt signaling pathway. Protein–protein interaction (PPI) network analysis identified the interaction between CEACAM5 and Galectin-9 that was also verified by molecular docking and co-IP assay. We further confirmed a reduction of CEACAM5 in SARS-CoV-2 spike stimulated enterocytes could promote the expression of Galectin-9 protein in CD4+T cells. Then it gave rise to the increasing release of inflammatory factors and increased apoptosis of CD4+T cells by inhibition of PI3K/AKT/mTOR pathway. Ultimately intestinal barrier dysfunction happened.

**Conclusion:**

Our results indicated that CEACAM5 overexpression and Galectin-9 knockdown played a protective role in intestinal barrier injury upon spike-Fc stimulation. Collectively, our findings identified firstly that SARS-CoV-2 spike induced intestinal barrier dysfunction through the interaction between CEACAM5 and Galectin-9. The result provides potential therapeutic targets in intestinal barrier dysfunction for treating severe COVID patients.

## 1 Introduction

The coronavirus disease 2019 (COVID-19), caused by Severe Acute Respiratory Syndrome Coronavirus-2 (SARS-CoV-2), has become one of the greatest global public health concerns since December 2019 (1). Most patients with COVID-19 are asymptomatic or present with mild symptoms (including fever, cough and fatigue), and a small proportion manifest with severe pneumonia even to death (2, 3). Besides pulmonary manifestations, gastrointestinal (GI) symptoms such as diarrhea, abdominal pain, vomiting, and anorexia have been found in COVID-19 patients and firstly reported by our team (4). Our previous study reported that over ten percents of patients simply presented with GI symptoms without any imaging features of COVID-19 pneumonia (4). We also provided molecular evidences with the detection of viral RNA in GI tissue (including esophagus, duodenum and rectum) and stool samples (5). Other studies have also highlighted the importance of GI symptoms in COVID-19 and correlated GI symptoms with disease severity and systemic inflammation (6, 7). During the infection of SARS-CoV-2, the spike proteins are cleaved and activated by transmembrane serine protease 2 (TMPRSS2) and furin (8), which interact with its cellular receptor, angiotensin-converting enzyme 2 (ACE2), to enter and infect host cells (9). Abundant ACE2 expression in GI tract may explain the GI symptoms in COVID-19 patients (10). Kuba et al. (11) found SARS-CoV spike RBD-Fc could bind to ACE2 and downmodulate ACE2 expression, and stimulation with SARS-CoV spike RBD-Fc worsened acid-induced acute lung injury in wild-type mice. As SARS-CoV-2 and SARS-CoV have too many similarities in genome, structure and et al., we have established an animal model mimicking intestinal inflammation upon stimulation with SARS-CoV-2 spike RBD-Fc protein and observed significant intestinal inflammation and co-localization of murine ACE2 with spike RBD-Fc in mice after the stimulation with spike RBD-Fc (12). However, the specific pathogenesis and mechanism of GI symptoms during SARS-CoV-2 infection remains poorly understood.

The intestinal mucosal barrier is mainly composed of mechanical, immune, microbial, and mucous barrier. It is the first defense to prevent intestinal microorganisms and bacterial toxins from entering the systemic circulation. It is found that expression of biomarkers of intestinal injury increased in urine (13) and plasm (14) of COVID-19 patients. Thus it is critical to maintain the integrity of intestinal mucosal barrier for the treatment of COVID-19 patients, especially with GI symptoms. Nevertheless, previous studies about the mechanism of intestinal barrier damage mainly focused on microbial dysbiosis. We and others have observed the altered gut microbiome in COVID-19 patients are characterized by beneficial gut bacteria reduction and opportunistic pathogen enrichment (15–17). Abnormal microbiota-host interplay can result in the disruption of gut epithelium barrier (18, 19). Thus, it’s necessary to further explore the regulatory network of intestinal barrier damage in SARS-CoV-2 infection, which might provide novel strategies to improve the therapeutic efficacy in COVID-19 patients.

Carcinoembryonic antigen-related cell adhesion molecule 5 (CEACAM5, also known as CEA or CD66e) was firstly reported as a tumor marker for colorectal cancer in 1965 (20). Besides, CEACAM5 has been demonstrated to modulate the systemic immune response through multiple pathways. CEACAM5 expressed on intestinal epithelial cells (IECs) can bind with CD8α on CD8+ suppressor T cells, leading to the inhibition of CD8+ suppressor T cell activation and the increasing proliferation of CD4+ T cells in inflammatory bowel disease (IBD) patients (21, 22). CEACAM5-derived peptide can activate CD8+ regulatory T cells to restore mucosal homeostasis (23). CEACAM5 mutation can inhibit TGF-β signaling and increase cell proliferation and colony formation in colorectal adenocarcinomas (24). Besides, high levels of CEACAM5 might increase the susceptibility of peripheral blood mononuclear cells (PBMCs) to Middle East respiratory syndrome-coronavirus (MERS-CoV) infection and promote disease progression (25). Through fecal multi-omics analysis, we found a decrease in CEACAM5 levels in COVID-19 patients (17). Moreover, expression of CEACAM5 was positively correlated with the abundance of Tyzzerella nexilis (beneficial gut bacteria), but negatively correlated with Bacteroides coprophilus (pathogenic gut bacteria) (17). However, the regulatory role and molecular mechanism of CEACAM5 in intestinal barrier dysfunction upon SARS-CoV-2 infection remains unclear.

Here we discussed the underlying working mechanisms of CEACAM5 in intestinal barrier dysfunction induced by SARS-Cov-2 spike, which may provide promising therapeutic targets for alleviating GI symptoms in COVID-19 patients and further enhance the treatment of SARS-Cov-2 infection.

## 2 Materials and methods

### 2.1 Animal experiments

All animal experiments were in accordance with the recommendations approved by the Experimental Animal Ethics Committee of the Fifth Affiliated Hospital of Sun Yat-sen University. C57BL/6J mice aged 7-8 weeks were purchased from Guangdong Medical Laboratory Animal Center (Guangdong, China). Mice models were established after one week of quarantine and acclimatization. After 24 hours of fasting, mice were anesthetized with isoflurane and administrated of 0.5ml acetic acid (1% vol/vol in saline) via enema 5cm proximal to the anus through a polyethylene catheter, held in an upside-down position for 2 minutes and then flushed with 0.5ml PBS enema. Experimental group was injected intraperitoneally with 5μg recombinant spike-Fc containing the receptor binding domain (RBD) (Sino Biological, 40592-V05H, diluted in 200ul PBS) after 16 hours, while control group with 5μg control -Fc (Sino Biological, 10690-MNAH-100, diluted in 200ul PBS). Mice were sacrificed after 6 hours, 2 days, 4 days and 7 days, and blood, intestine and colon samples were collected. A part of the intestine and colon tissues were fixed with 4% paraformaldehyde for histopathological staining, and the remnants were stored at –80°C for subsequent use.

### 2.2 Hematoxylin–eosin, immunohistochemistry, and immunofluorescence staining

Intestine and colon tissues were fixed with 4% paraformaldehyde, embedded in paraffin, and stained with hematoxylin-eosin (HE) following standard protocol for histopathological analysis. Immunohistochemistry was performed to determine the protein expression of CEACAM5. Briefly, tissue sections were blocked with blocking buffer (Beyotime, P0260) for 30 minutes at room temperature. Then slides were incubated overnight at 4°C with the primary antibodies of CEACAM5 (Abclonal, A12421). After rinsing with PBS, slides were incubated for 30 min at 37°C with HRP Polymer Conjugate (ZSGB-BIO) and observed by microscopy.

Immunofluorescence was used to determine the degree of proliferation in CD4+ T cells. After blocked as previously described, slides were incubated overnight at 4°C with the primary antibodies of CD4 (Servicebio, GB13064-2) and Ki67 (Servicebio, GB111141). The slides were incubated with secondary antibodies (Alexa Fluor^®^647-conjugated goat anti-rabbit IgG, bs-0296G-AF647; Dylight-550 Goat Anti-rabbit IgG secondary antibody, BA1135) for 1 h at room temperature followed by washing three times with PBST. After counterstaining nuclei with 4’,6-diamidino-2-phenylindole (DAPI), slides were imaged using a fluorescence microscopy (Nikon Eclipse C1). Staining results were evaluated by two independent observers.

### 2.3 Histological evaluation of the intestine tissues from mice

Histological evaluation was performed by the pathologists and the severity of inflammation was determined as previous described (26). Negative, lack of lesions; mild inflammation, scattered leukocyte infiltration in lamina propria, increased height of proliferating crypts; moderate inflammation, multifocal aggregates of infiltrating leukocytes in lamina propria extending into the submucosa, increased height, and proliferation of mucosa with loss of goblet cells, crypt abscesses detectable; severe inflammation, coalescing aggregates of infiltrating leukocytes expanding lamina propria and submucosa with evidence of crypt dropout.

### 2.4 Western blotting and co-IP

Total protein extraction and western blotting were performed as described previously (12). The primary antibodies included CEACAM5 (Abclonal, A12421, 1:1000), Galectin-9 (Origene, TA805651S, 1:1000), ZO-1 (Proteintech, 21773-1-AP, 1:1000), PI3K (Beyotime, AF7749, 1:1000), mTOR (Beyotime, AM832, 1:1000), p-mTOR (Beyotime, AF5869, 1:1000), AKT (Beyotime, AA326, 1:1000), and p-AKT (Beyotime, AA329, 1:1000). Also, the secondary antibodies, including anti-rabbit IgG (H+L) and anti-mouse IgG (H+L), were purchased from Proteintech (1:5000). ImageJ software was used to analyze the protein bands. For co-IP, lysates of mice intestinal tissues were immunoprecipitated with IP buffer containing IP antibody-coupled agarose beads, and protein complexes were later subjected to western blotting, while IgG was used as a negative control.

### 2.5 Cell culture and treatments

Human colonic adenocarcinoma cell line Caco-2 was cultured in Dulbecco’s modified Eagle’s medium (DMEM, Gibco, USA) supplemented with 10% fetal calf serum (FCS, Gibco, USA), 1% penicillin/streptomycin mixture, and incubated at 37°C containing 5% CO2. CEACAM5-shRNA expressing lentiviruses (sh-CEACAM5), CEACAM5 overexpression plasmid and Galectin-9 siRNA plasmids were bought from GeneCopoeia (Guangzhou, China).

### 2.6 Elisa assay

Serum was collected from 18 severe COVID-19 patients and 18 non-severe COVID-19 patients, 17 spike-Fc RBD mice and 17 control-Fc mice, and serum soluble LPS levels were measured using a human/mouse LPS ELISA Kit (Cloud-Clone Corp, SEB526Ge).

### 2.7 CD4+ T cell isolation, co-cultured with Caco-2 and flow cytometry analysis

Blood was collected from healthy donors, and peripheral blood mononuclear cells (PBMC) were separated by density gradient centrifugation. Then, CD4+ T cells were isolated with magnetic beads (Biolegend, 480009). The Caco-2 cells were plated in a 6-well plate using RPMI 1640 medium (Gibco, USA) one day ahead. After removing non-adherent cells by washing and changing with fresh medium, CD4+ T cells were added. After 48h, the cell medium was collected and centrifuged to collect CD4+ T cells, while Caco-2 cells were collected after trypsin digestion. Immune cell number and apoptosis were assessed by a CytoFLEX LX flow cytometer (Beckman Coulter) according to the manufacturer’s instructions.

### 2.8 RNA isolation and qRT-PCR

Total RNA was extracted from samples using an RNA extraction kit (Vazyme, Nanjing, China). Reverse transcription kits (Vazyme, Nanjing, China) were used for reverse transcription of RNA into cDNA. Quantitative real-time PCR (qRT-PCR) was conducted with ChamQ Universal SYBR qPCR Master Mix (Vazyme, Nanjing, China) on Real-Time PCR Detection System (Bio-Rad). The primers designed by Guangzhou IGE Biotechnology (Guangzhou, China) were as indicated in **Supplementary Table 1**.

### 2.9 Statistical analysis

Statistical analyses were performed using GraphPad PRISM software v8.4.2. All experiment was repeated three or more times, and experimental data were shown as mean ± standard deviation (SD). The experimental data were tested for homogeneity of variances and normality. Comparisons between two groups were analyzed using Students t-test, and one-way ANOVA was applied in comparisons between multiple groups with homogeneous variance while non-parametric test (The Mann-Whitney test) was used for inhomogeneity. P < 0.05 were considered statistically significant (*p < 0.05, **p < 0.01, ***p < 0.001).

## 3 Results

### 3.1 Decreased CEACAM5, tight junction protein and CD4+ T cells and increased inflammatory factors in intestinal tissue after the stimulation with SARS-CoV-2 spike RBD-Fc

**Figure 1:**
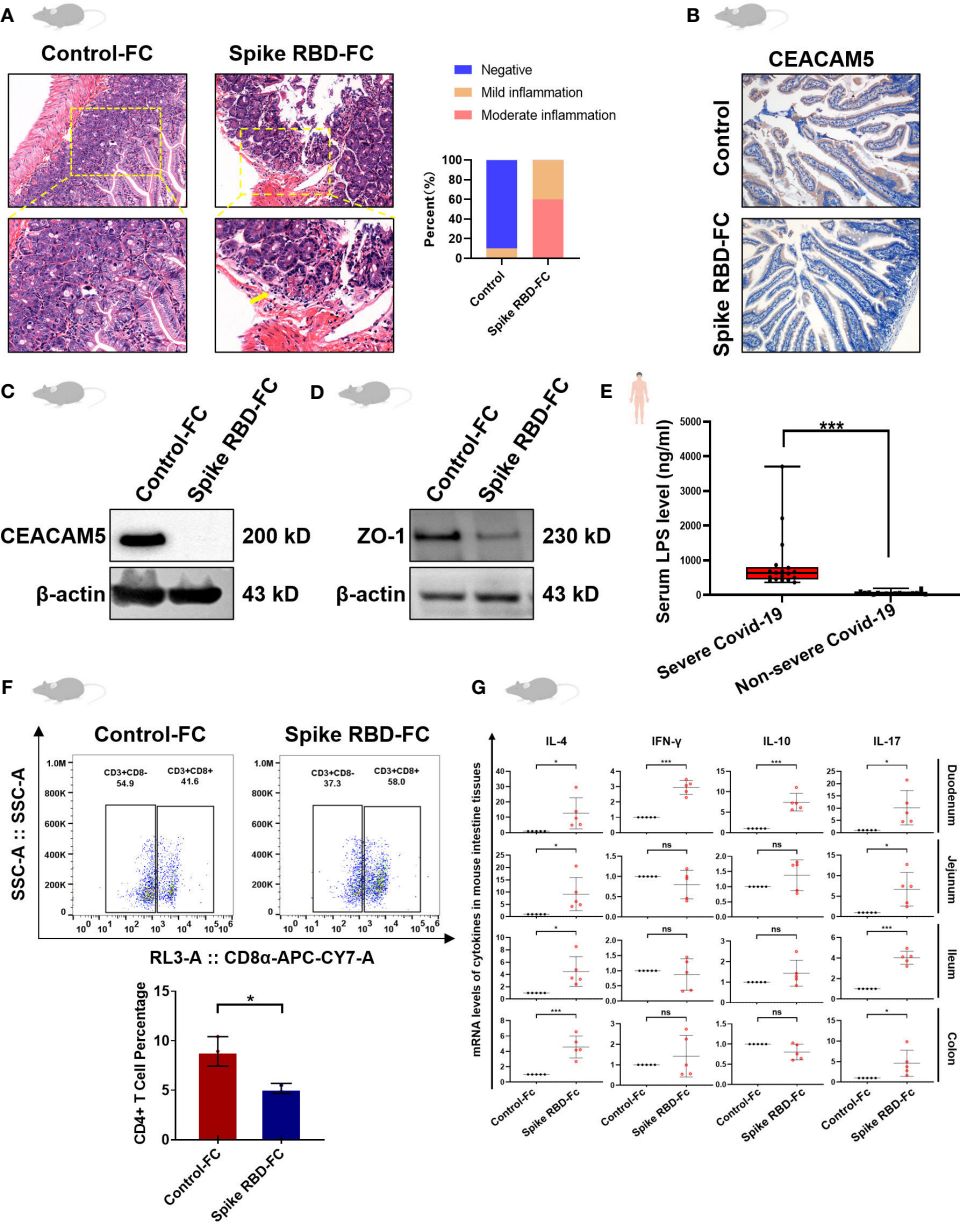
Decreased CEACAM5, intestine barrier loss and abnormal intestinal immune function after the stimulation with SARS-CoV-2 spike RBD-Fc. **(A)** Representative HE images and the quantitative analysis of inflammation of the duodenum from mice, and marked the areas of edema and inflammatory exudation with yellow arrows under the high-power field of view. **(B)** Immunohistochemical staining and **(C)** western blotting showed the reduced CEACAM5 expression in intestines of mice after spike RBD-Fc stimulation. **(D)** Western blotting showed low expression of ZO-1 in intestines of mice after spike RBD-Fc stimulation. **(E)** ELISA found high levels of LPS in severe COVID-19 patients compared to control. **(F)** Flow cytometric analysis showed the reduced CD4+ T cells in intestines of mice after spike RBD-Fc stimulation (n=5), and the difference of the ratio of CD4+ T cells to total lymphocytes between spike RBD-FC group and control-FC group using bar graphs. **(G)** Overexpression of cytokines in intestines of mice after spike RBD-Fc stimulation. Data were shown as the mean ± SD. *p < 0.05; **p < 0.01; ***p < 0.001. ns, no significance.

To further validate the reduced CEACAM5 expression in feces of COVID-19 patients (17) and explore its biological functions, mice models were established through intraperitoneally injecting recombinant viral spike-Fc containing RBD to mimic the intestinal inflammation (**Figure 1A**) as we previously reported (12). As expected, the expression of CEACAM5 in intestine of spike RBD-Fc group decreased. (**Figure 1B, C**). We further examined the intestinal barrier, and found zona occludens 1 (ZO-1) expression in intestine was decreased in spike RBD-Fc stimulated mice compared with control (**Figure 1D**), which was consistent with the increased level of LPS, a plasma biomarker of intestinal barrier injury, in severe COVID-19 patients compared with non-severe ones (**Figure 1E**, p < 0.01). To study the local immune responses, we isolated and counted the mononuclear cells from mice intestinal tissues. CD4+T cells of spike RBD-Fc stimulated mice intestine decreased significantly (**Figure 1F**, p<0.05). Furthermore, we detected the expression level of CD4+ T cells activation-related inflammatory factors and discovered the level of IL-4, INF-γ, IL-10 and IL-17 were increased with varying degrees in mice intestine after spike RBD-Fc stimulation (**Figure 1G**). There was a marked increase of inflammatory factors in duodenum (**Figure 1G**), which is consistent with the typical pathological manifestations of duodenum. Together, these data indicated the potential role of CEACAM5 in intestinal barrier and immune responses upon SARS-CoV-2 spike RBD-Fc stimulation.

### 3.2 Proteomic analysis of intestinal tissues from mice stimulated with SARS-CoV-2 spike RBD-Fc

**Figure 2:**
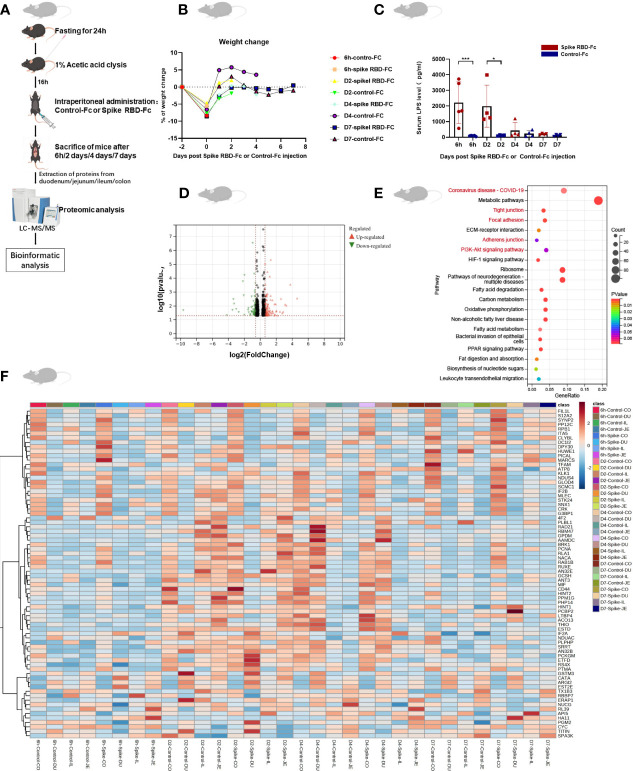
Proteomic analysis of intestinal tissues from mice stimulated with SARS-CoV-2 spike RBD-Fc. **(A)** Illustration of the establishment of animal models and proteomic analysis. **(B)** Weight change of mice during the experimental period. **(C)** ELISA showed higher level of LPS in plasm of mice at 6 hours and 2 days after spike RBD-Fc stimulation. **(D)** Volcano plot showed all differentially expressed proteins. **(E)** KEGG enrichment analysis of differential expressed proteins. **(F)** Heatmap of differentially expressed proteins. Data were shown as the mean ± SD. *p < 0.05; **p < 0.01; ***p < 0.001.

To decipher the functional mechanisms of CEACAM5 after spike RBD-Fc stimulation, we performed proteomic analysis on intestine tissues (including duodenum, jejunum, ileum, and colon) in spike RBD-Fc stimulated mice models and control group (**Figure 2A**). During the experimental period, significant loss of body weight was observed in all mice without marked difference between spike and control groups (**Figure 2B**). We further evaluated the plasma levels of LPS in mice (n=34) by ELISA. Mice in spike group had a higher level of LPS at 6 hours and day 2, which indicated intestinal barrier dysfunction in mice at 6 hours and 2 days after spike RBD-Fc stimulation (**Figure 2C**). Among 1985 proteins identified, a total of 549 differentially expressed proteins (fold change > 1.2 and p < 0.05) were detected in intestine at four different time points (**Figure 2D**). KEGG analysis revealed that differentially expressed proteins were mainly enriched in COVID-19, tight junction, focal adhesion, adherens junction and PI3K-Akt signaling pathway (**Figure 2E**). Besides, we used heatmaps to further display differentially expressed proteins on intestine tissues between spike RBD-Fc stimulated mice models and control group (**Figure 2F**).

### 3.3 Increased Galectin-9 expression after the stimulation with SARS-CoV-2 spike RBD-Fc and the interaction between CEACAM5 and Galectin-9

**Figure 3:**
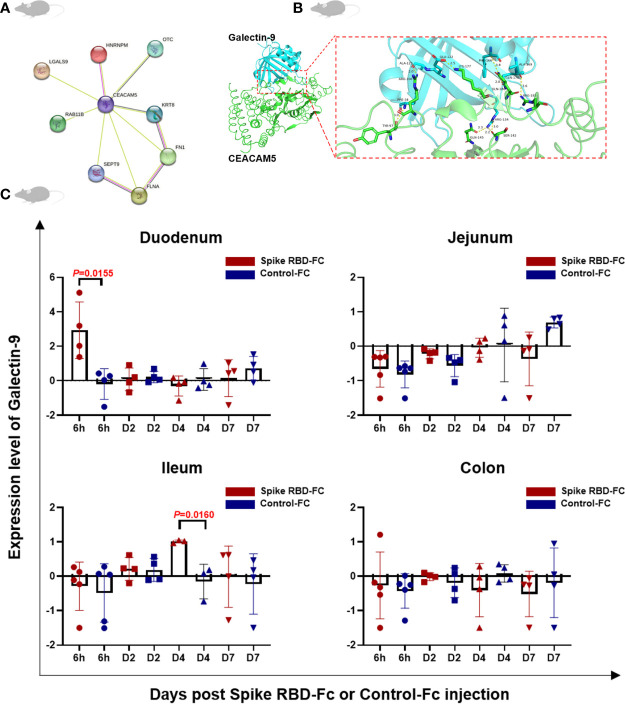
Increased Galectin-9 expression after the stimulation with SARS-CoV-2 spike RBD-Fc and the interaction between CEACAM5 and Galectin-9. **(A)** Protein–protein interaction network of CEACAM5 showed that top 8 potential proteins in differentially expressed proteins might interact with CEACAM5. **(B)** Molecular docking confirmed the interaction between CEACAM5 (green) and Galectin-9 (blue). **(C)** The increased expression of Galectin-9 in duodenum at 6 hours and ileum at 4 days after spike RBD-Fc stimulation. Data were shown as the mean ± SD.

We screened out eight differentially expressed proteins having potential interactions with CEACAM5 through the protein-protein interaction network analysis based on STRING database (**Figure 3A**). Among them, Galectin-9 (Gal-9, LGALS9, GALECTIN-9) is a ligand to immune checkpoint protein TIM-3, which expresses in variety of immune cells and regulates a multitude of cellular processes. Molecular docking further confirmed the interaction between CEACAM5 and Galectin-9 (**Figure 3B**). Our proteomic results revealed that the expression of Galectin-9 was significantly increased in duodenum at 6 hours and ileum at 4 days after the stimulation with SARS-CoV-2 spike RBD-Fc (**Figure 3C**). The result indicates the interaction between CEACAM5 and Galectin-9 might play an important role in enteric stimulation of spike RBD-Fc and impaired intestinal barrier function.

### 3.4 Inhibiting PI3K/Akt/mTOR pathway and CD4+ T cells proliferation in mice tissues stimulated by SARS-Cov-2 spike RBD-Fc

**Figure 4:**
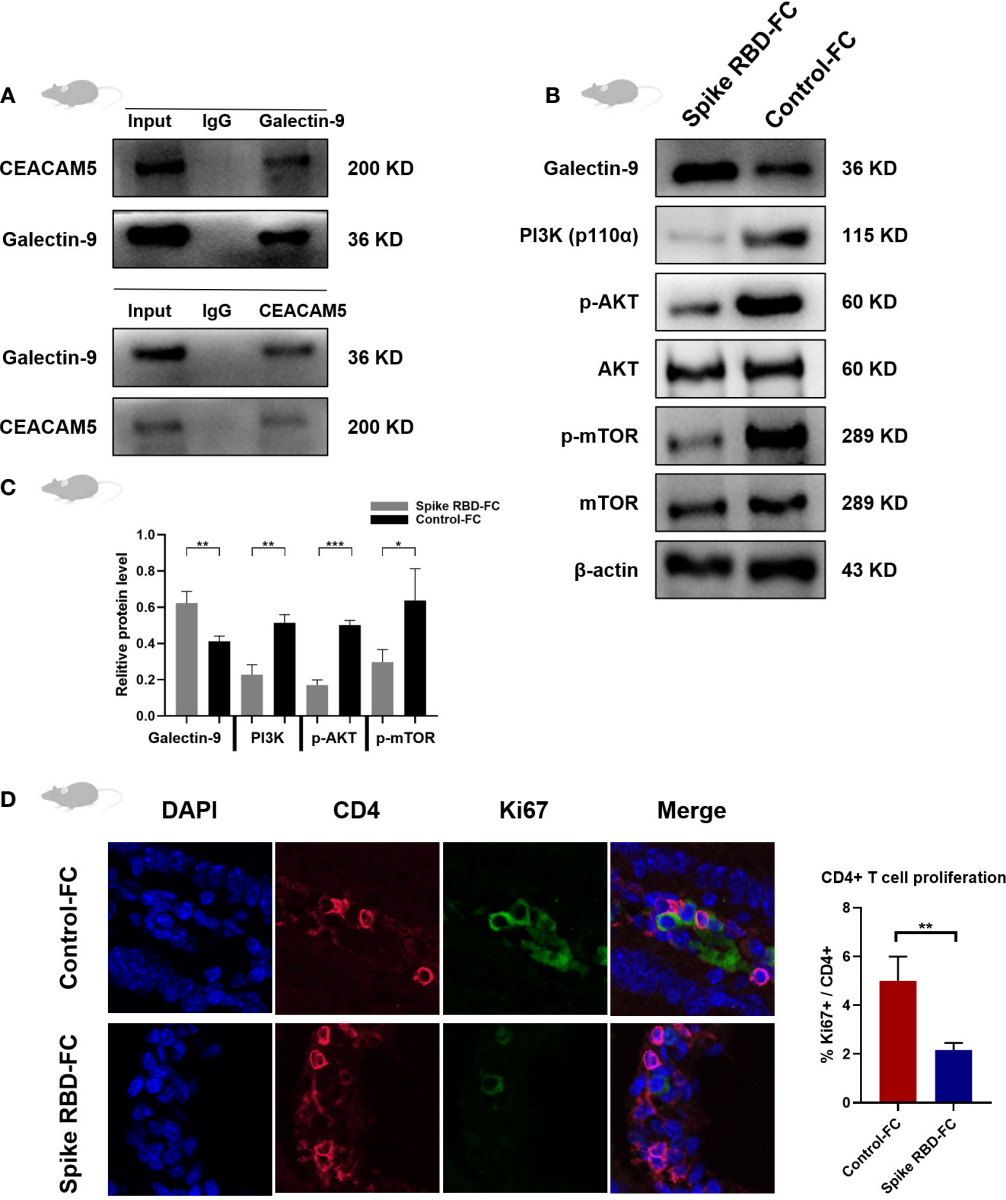
Inhibiting PI3K/Akt/mTOR pathway and CD4+ T cells proliferation in mice tissues stimulated by SARS-Cov-2 spike RBD-Fc. **(A)** Co-IP assay verified the bindings between CEACAM5 protein and Galectin-9 protein in intestine tissues from mice. **(B)** Representative protein bands and **(C)** quantification analyses of the expression of Galectin-9, PI3K, p-AKT and p-mTOR in intestine tissues from mice. **(D)** Immunofluorescence result of CD4 and Ki67 in intestine tissues from mice. *p < 0.05; **p < 0.01; ***p < 0.001.

To further explore the mechanism of intestinal barrier damage during SARS-CoV-2 spike RBD-Fc stimulation, co-IP and western blotting were conducted to verify the interaction between CEACAM5 and Galectin-9 in mice intestine tissues and found CEACAM5 may bind with Galectin-9 (**Figure 4A**). We found the significantly increased protein levels of Galectin-9, and decreased expression of PI3K, p-AKT and p-mTOR in spike RBD-Fc group intestine tissues compared to control (**Figure 4B, C**, p < 0.05). The result indicates the inhibition of PI3K/AKT/mTOR signaling pathway in intestine after the stimulation with SARS-CoV-2 spike RBD-Fc. Besides, we observed the expression of Ki67 was decreased in CD4+ T cells from spike RBD-Fc stimulated mice intestine using CD4 and Ki67 double immunofluorescence staining (**Figure 4D**), which indicates the inhibited proliferation of CD4+ T cells after spike RBD-Fc stimulation.

### 3.5 SARS-Cov-2 spike RBD-Fc induced intestinal barrier damage through the interaction between CEACAM5 and Galectin-9

**Figure 5:**
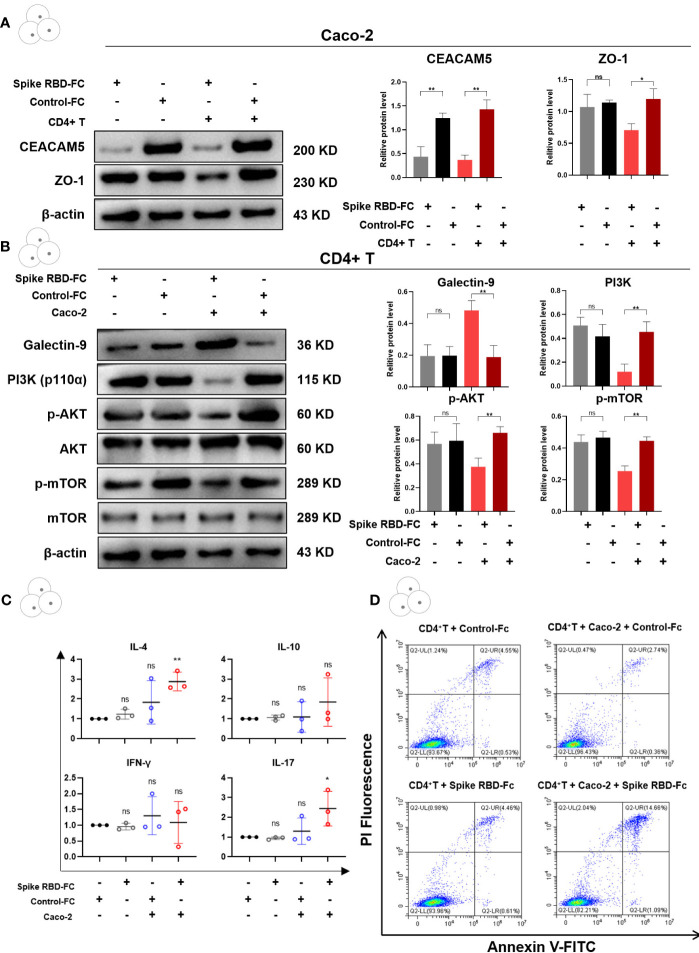
SARS-Cov-2 spike RBD-Fc induced intestinal barrier damage through the interaction between CEACAM5 and Galectin-9. **(A)** Representative protein bands and quantification analyses of the expression of CEACAM5 and ZO-1 in Caco-2 cells. **(B)** Representative protein bands and quantification analyses of the expression of Galectin-9, PI3K, p-AKT and p-mTOR in CD4+ T cells. **(C)** The mRNA levels of cytokine in CD4+ T cells by qPCR. **(D)** The detection of apoptotic CD4+ T cells by flow cytometry. Data were shown as the mean ± SD.*p < 0.05; **p < 0.01; ***p < 0.001.ns, no significance.

Spike RBD-Fc stimulation model of intestinal epithelial cells was constructed using spike RBD-Fc and Caco-2 cells. Western blotting analysis revealed a significant decrease in the levels of CEACAM5 protein in Caco-2 cells after spike RBD-Fc stimulation (**Figure 5A**, p < 0.05), but there was no difference of ZO-1 among the groups (**Figure 5A**, p > 0.05). Then, CD4+ T cells isolated from peripheral blood of healthy humans were cocultured with Caco-2. We found spike RBD-Fc stimulation induces significant downregulation of ZO-1 in addition to a decrease of CEACAM5 in Caco-2 cells (**Figure 5A**, P<0.05). These results suggested that SARS-CoV-2 spike RBD-Fc caused intestinal barrier damage through the interaction between Caco-2 and CD4+ T cells.

As the protein levels of Galectin-9, PI3K, p-AKT and p-mTOR in CD4+ T cells showed no difference between spike RBD-Fc and control-Fc group (**Figure 5B**, p > 0.05), we observed a significant elevation of Galectin-9, and decreased expression of PI3K, p-AKT and p-mTOR in spike RBD-Fc group after the coculture with Caco-2 cells (**Figure 5B**, p < 0.05). Furthermore, we examined the cytokine levels of CD4+ T cells by quantitative real-time PCR, and found that the mRNA expression of IL-4 and IL-17 was significantly increased only in spike RBD-Fc + Caco-2 + CD4+ T group (**Figure 5C**, p < 0.05). As expected, flow cytometric analysis showed a significantly higher proportion of apoptotic cells in CD4+ T cells when cocultured with Caco-2 cells and spike RBD-Fc (**Figure 5D**). Taken together, these findings indicated that the spike RBD-Fc stimulation of enterocytes downregulated the expression of CEACAM5 protein, and upregulated Galectin-9 expression in CD4+ T cells through the interaction between CEACAM5 and Galectin-9. Then the polarization of CD4+ T cells towards pro-inflammatory was induced, inhibiting the PI3K/AKT/mTOR pathway and causing increased apoptosis of CD4+ T cells. Eventually the intestinal barrier damage developed.

### 3.6 CEACAM5 knockdown in intestinal epithelial cell upregulated Galectin-9, inhibited the PI3K/AKT/mTOR pathway in CD4+ T cells, and damaged intestinal barrier

**Figure 6:**
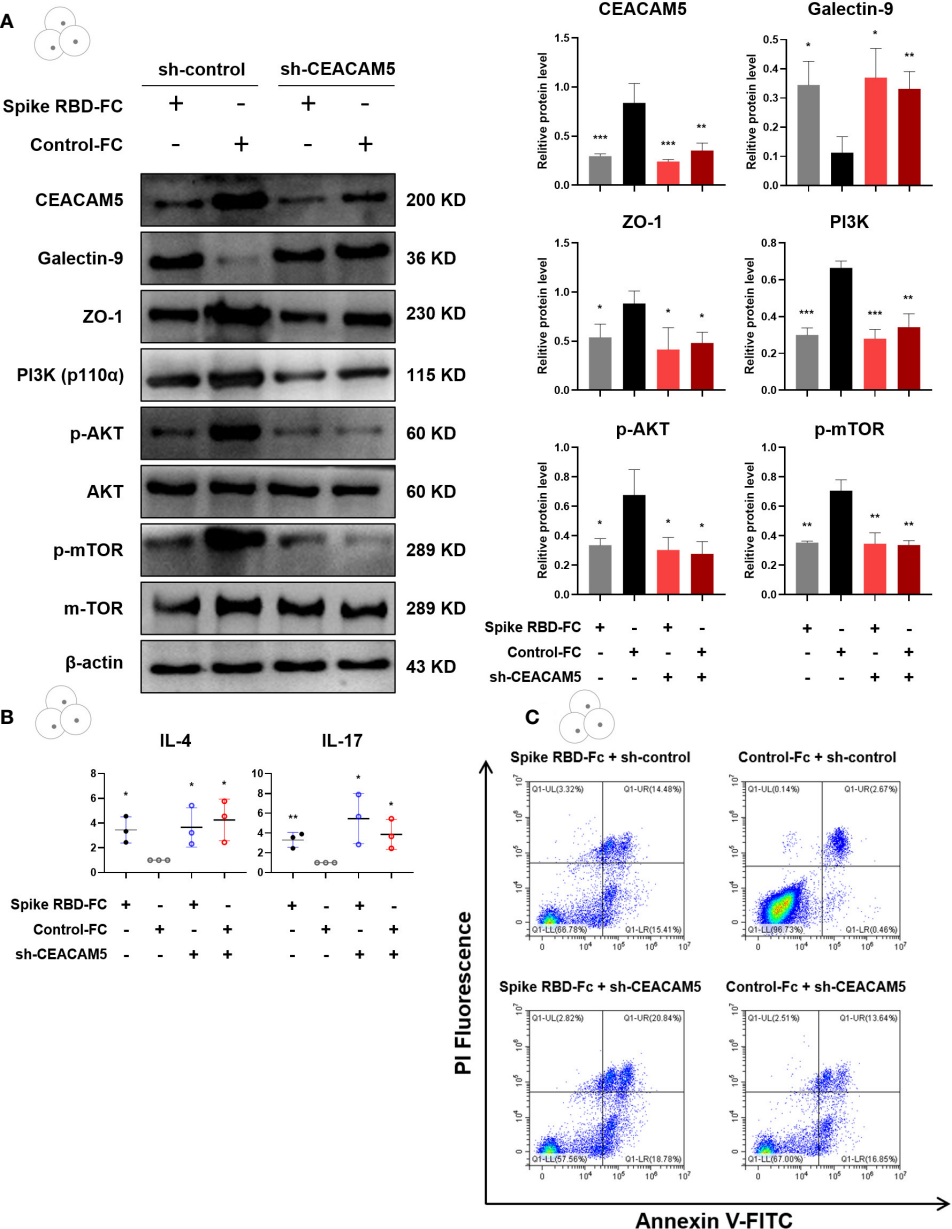
CEACAM5-knockdown in intestinal epithelial cell upregulated Galectin-9, inhibited the PI3K/AKT/mTOR pathway in CD4+ T cells, and damaged intestinal barrier. **(A)** Representative protein bands and quantification analyses of CEACAM5, Galectin-9, ZO-1, PI3K, p-AKT, p-mTOR in Caco-2 cells transfected with CEACAM5-shRNA lentiviruses. **(B)** The mRNA levels of cytokine in CD4+ T cells by qPCR. **(C)** The detection of apoptotic CD4+ T cells by flow cytometry. Data were shown as the mean ± SD.*p < 0.05; **p < 0.01; ***p < 0.001.

In order to confirm the role of CEACAM5 in intestinal barrier injury, we respectively transfected Caco-2 cells with CEACAM5 overexpression plasmid and CEACAM5-shRNA expressing lentiviruses (sh-CEACAM5). Empty plasmid and control lentivirus served as control groups respectively. The expression of Galectin-9 in CD4+ T cells was significantly increased (p < 0.05), while the expression of CEACAM5 and ZO-1 in Caco-2 cells (all p < 0.05), PI3K, p-AKT and p-mTOR in CD4+ T cells (all p < 0.05) were remarkably decreased in the group of Caco-2 cells transfected with sh-CEACAM5 (**Figure 6A**). Similarly, CEACAM5-knockdown could increase the inflammatory factors IL-4 and IL-17 secreted by CD4+ T cells (**Figure 6B**). Furthermore, both CEACAM5-knockdown and spike RBD-Fc stimulation increased the apoptosis of CD4+ T cells (**Figure 6C**). These data suggested that the downregulation of CEACAM5 could damage intestinal barrier, which was independent of spike RBD-Fc stimulation. And the decrease of CEACAM5 after spike RBD-Fc stimulation subsequently induced intestinal barrier dysfunction by increasing the expression of Galectin-9 and inducing the polarization of CD4+ T cells towards pro-inflammatory phenotype and increased apoptosis.

### 3.7 Overexpression of CEACAM5 in intestinal epithelial cells protected against barrier damage after spike RBD-Fc stimulation

**Figure 7:**
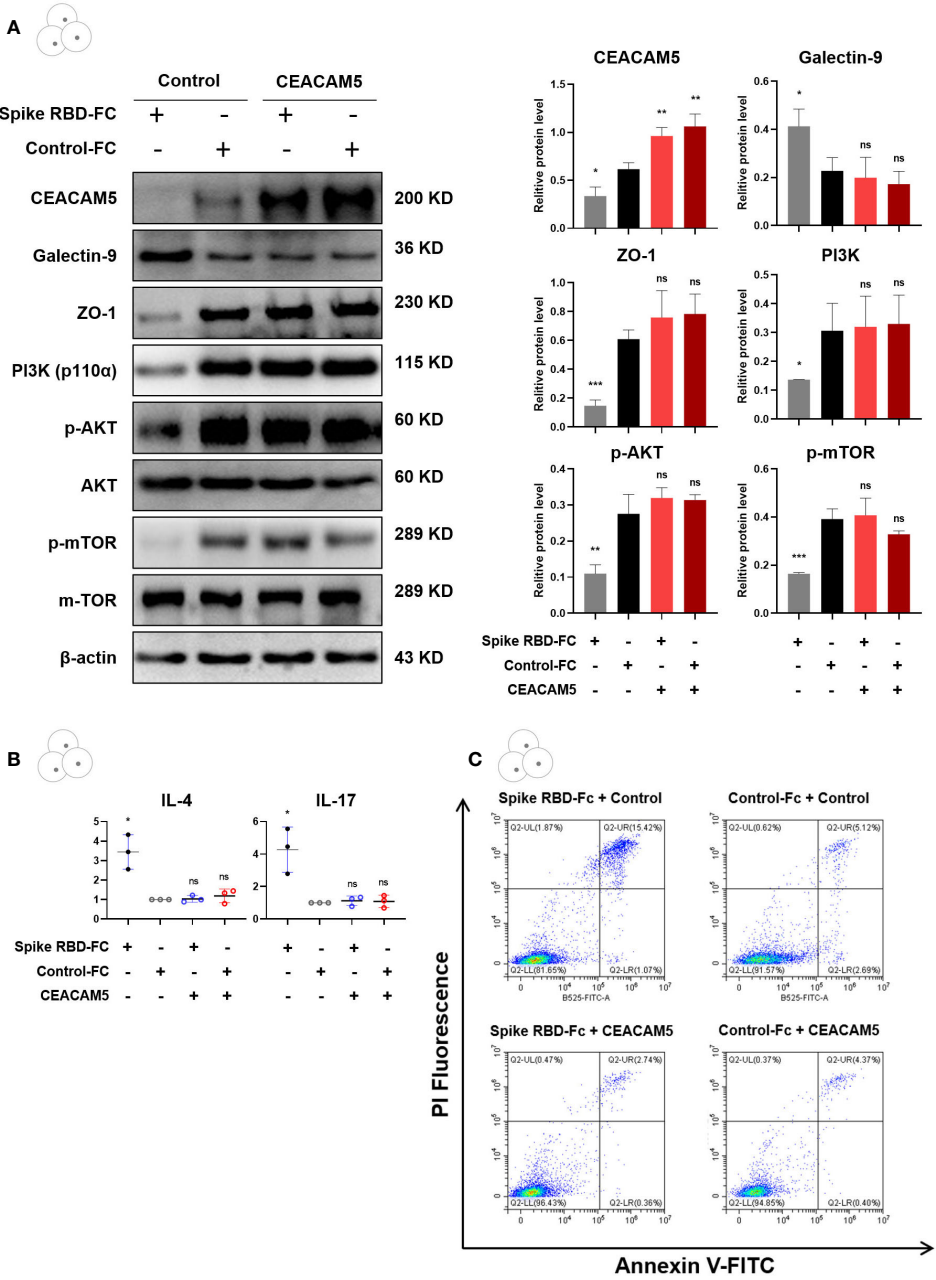
Overexpression of CEACAM5 in intestinal epithelial cells protected against barrier damage after spike RBD-Fc stimulation. **(A)** Representative protein bands and quantification analyses of CEACAM5, Galectin-9, ZO-1, PI3K, p-AKT, p-mTOR in Caco-2 cells transfected with CEACAM5 overexpression plasmid. **(B)** The mRNA levels of cytokine in CD4+ T cells by qPCR. **(C)** The detection of apoptotic CD4+ T cells by flow cytometry. Data were shown as the mean ± SD.*p < 0.05; **p < 0.01; ***p < 0.001. ns, no significance.

The expression of Galectin-9 in CD4+ T cells was significantly decreased (p < 0.05), while the expression of CEACAM5 and ZO-1 in Caco-2 cells (all p < 0.05), PI3K, p-AKT, p-mTOR in CD4+ T cells (p < 0.05) were remarkably increased in the group of Caco-2 cells transfected with CEACAM5 overexpression plasmid (**Figure 7A**). Moreover, the overexpression of CEACAM5 completely reversed the elevation of cytokines IL-4 and IL-17 secreted by CD4+ T cells after spike RBD-Fc stimulation (**Figure 7B**). For increased apoptosis upon spike RBD-Fc stimulation, CEACAM5 overexpression groups with or without spike RBD-Fc, and control group without spike RBD-Fc all presented a lower proportion of apoptotic CD4+ T cells than control group with spike RBD-Fc (**Figure 7C**). The above results indicated that the overexpression of CEACAM5 could reverse spike RBD-Fc induced increase of Galectin-9, the polarization of CD4+ T cells towards pro-inflammatory phenotype, the inhibition of PI3K/AKT/mTOR pathway and increased apoptosis of CD4+ T cells. Thus, CEACAM5 might serve as a protective factor in intestinal epithelial injury upon spike RBD-Fc stimulation.

### 3.8 Galectin-9-knockdown inhibited the intestinal barrier damage induced by CEACAM5 downregulation after spike RBD-Fc stimulation

**Figure 8:**
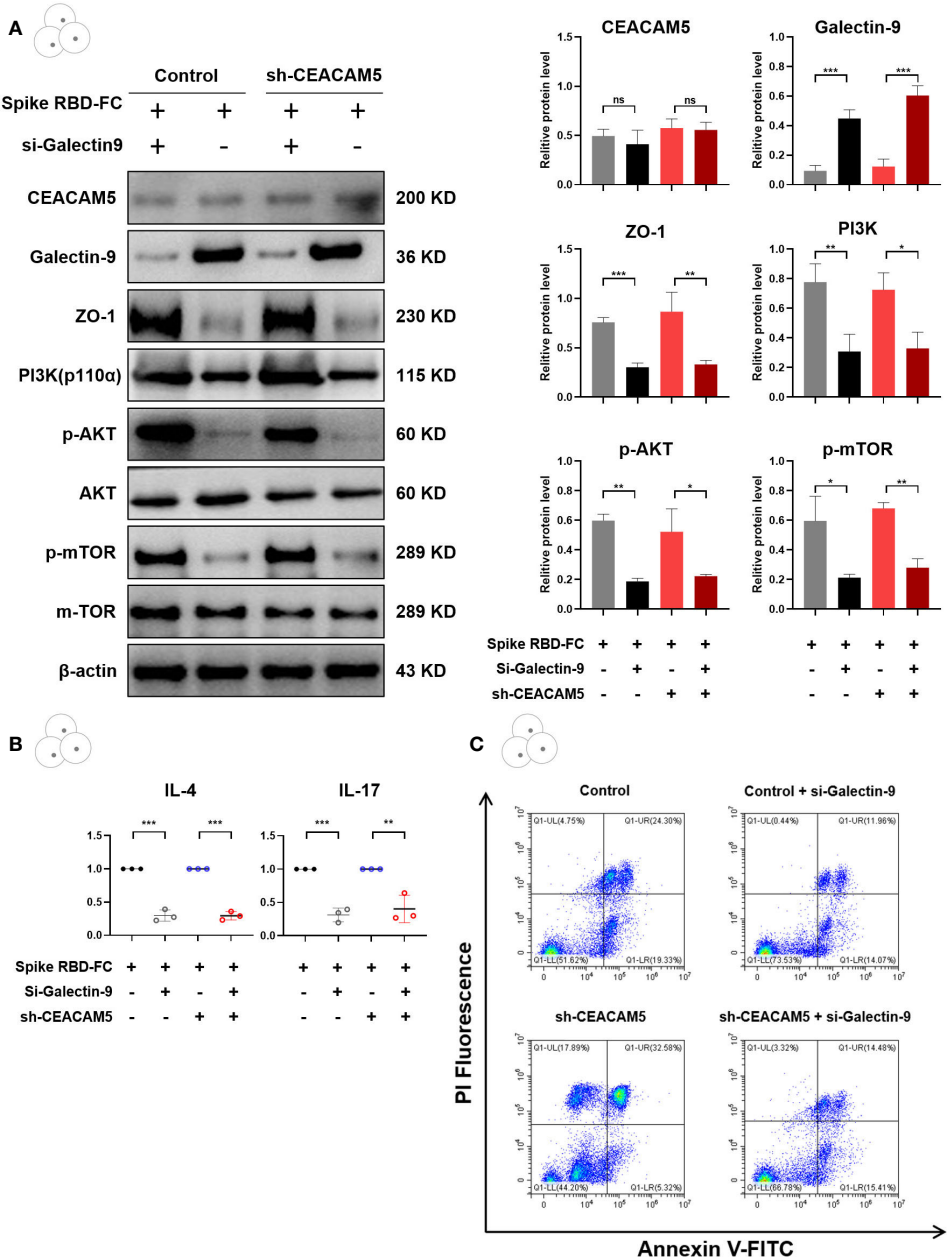
Galectin-9-knockdown inhibited the intestinal barrier damage induced by CEACAM5 downregulation after spike RBD-Fc stimulation. **(A)** Representative protein bands and quantification analyses of CEACAM5, Galectin-9, ZO-1, PI3K, p-AKT, p-mTOR in CD4+ T cells transfected with Galectin-9 siRNA plasmids. **(B)** The mRNA levels of cytokine in CD4+ T cells by qPCR. **(C)** The detection of apoptotic CD4+ T cells by flow cytometry. Data were shown as the mean ± SD.*p < 0.05; **p < 0.01; ***p < 0.001. ns, no significance.

To further dissect the regulatory role of Galectin-9 in spike RBD-Fc induced barrier damage, Galectin-9 knockdown was conducted in CD4+ T cells through transfecting Galectin-9 siRNA plasmids. Caco-2 cells transfected with control lentivirus or sh-CEACAM5 lentiviruses were treated with spike RBD-Fc. CD4+ T cells transfected with control siRNA or Galectin-9 siRNA plasmids were added to co-culture systems. CD4+ T cells with Galectin-9-knockdown showed a significantly increased levels of ZO-1 in Caco-2 cells (p < 0.05), PI3K, p-AKT and p-mTOR in CD4+ T cells (all p < 0.05) compared to control siRNA group (**Figure 8A**). Similarly, Galectin-9-knockdown groups showed significantly lower expression of cytokines IL-4 and IL-17 after spike RBD-Fc stimulation (**Figure 8B**). In addition, Galectin-9-knockdown alleviated the apoptosis of CD4+ T cells compared to control siRNA group (**Figure 8C**). Taken together, the increase of Galectin-9 is essential in intestinal barrier damage after spike RBD-Fc stimulation, which might provide novel stratifies for GI symptoms in COVID-19 patients.

## 4 Discussion

**Figure 9:**
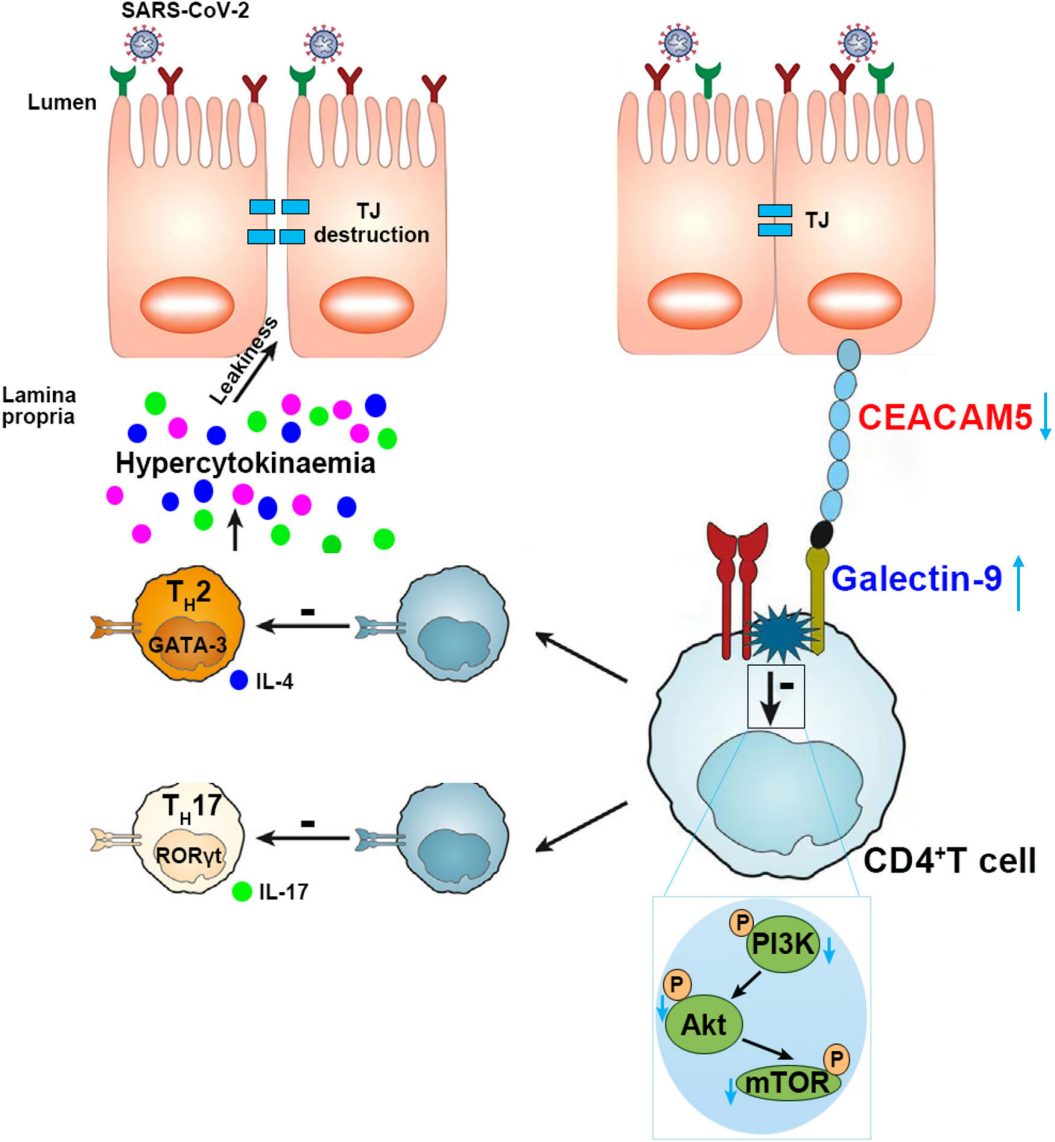
Mechanism diagram summarized that SARS-Cov-2 spike induced intestinal barrier dysfunction through the interaction between CEACAM5 and Galectin-9. SARS-CoV-2 spike reduced CEACAM5 protein expression in infected enterocytes, promoted Galectin-9 protein expression in CD4+ T cells through the interaction between CEACAM5 and Galectin-9, promoted the polarization of CD4+ T cells towards pro-inflammatory phenotype and increased apoptosis, eventually leading to intestinal barrier dysfunction.

In this study, we uncovered the low expression and protective role of CEACAM5 in intestinal barrier dysfunction induced by SARS-Cov-2 spike. CEACAM5 acted as a protective protein in maintaining intestinal barrier homeostasis in normal physiological states through binding to Galectin-9 and inhibiting Galectin-9 expression and promoting PI3K/Akt/mTOR pathways activation in CD4+ T cells. Therefore, when the expression of CEACAM5 is reduced after the stimulation with SARS-Cov-2 spike, its protective effect on intestinal barrier homeostasis is also reduced. Thus reduced CEACAM5 protein expression in enterocytes could increase Galectin-9 protein expression and inhibit PI3K/Akt/mTOR pathways in CD4+ T cells. Then inflammatory factors released and increased apoptosis of CD4+ T cells happened and eventually intestinal barrier dysfunction developed (**Figure 9**). CEACAM5 overexpression and Galectin-9 knockdown could relieve the intestinal barrier dysfunction stimulated by SARS-Cov-2 spike. This study illuminated the molecular mechanism of CEACAM5 in intestinal barrier dysfunction induced by SARS-Cov-2 spike, providing potential therapeutic strategies to alleviate intestinal barrier damage in severe COVID-19 patients.

GI symptoms are common extrapulmonary manifestations of SARS-CoV-2 infection. Researchers have revealed that SARS-CoV-2 can infect GI in human-derived intestinal organoids (27, 28), experimental animal models such as nonhuman primate model (29, 30) and Syrian hamster (31, 32). Moreover, severe COVID-19 has been associated with high levels of biomarkers in intestinal barrier disruption (33). But little is known about the pathogenesis of impaired intestinal barrier in SARS-CoV-2 infection. As enterocytes are main target cells of SARS-CoV-2, viral infection of enterocytes is the first and crucial step in SARS-CoV-2 induced gut immunological changes. Many studies have reported the changes in immune cells in the gastrointestinal tissues of patients infected with SARS-CoV-2, including the dysregulation of CD4+ T cells (34) and overactivated production of IL-17 from Th17 cells (35). Besides, Li et al. found increased production of IL-4, IL-17A and other inflammatory phenotype in gastrointestinal tissues of rhesus monkeys after intranasal infection with SARS-CoV-2 (36). Our findings firstly revealed that reduced CEACAM5 in enterocytes upon spike stimulation could induce immune abnormalities thus leading to intestinal barrier injury. And further studies are needed to explore the protective role of CEACAM5 in intestinal barrier injury especially in severe COVID-19 patients.

As a conventional tumor marker of colorectal cancer, CEACAM5 plays an important role in multiple tumors. Recently, its immunomodulatory effects have drawn increasing attention. The researchers elucidated that CEACAM5 activated CD8+ suppressor T cells through its B3 domain interacting with CD1d and N domain binding to CD8α (22). The deficiency of CEACAM5 in IBD patients inhibited CD8+ suppressor T cells activation, which led to the failure of suppressing CD4+ Th cell activation, thus resulting in pro-inflammatory factor release and inflammation progression. However, its pathogenic role in intestinal barrier injury especially caused by SARS-CoV-2 has not been explored. We previously observed the decreased expression of host CEACAM5 protein in COVID-19 patients feces (17). We confirmed the reduced CEACAM5 upon spike stimulation in mice and cell models, and found that the downregulation of CEACAM5 induced immune abnormalities of CD4+ T cells (including the polarization of CD4+ T cells towards pro-inflammatory phenotype and increased apoptosis) through the interaction between CEACAM5 and Galectin-9. Besides, the mechanism underlying the decreased expression of CEACAM5 remains to be explored. Previous studies have found that transcription factor sex determining region Y-box 9 (SOX9) was closely related to the expression of CEACAM5, and SOX9 downregulated CEACAM5 gene expression in human colon carcinoma cell line HT29Cl.16E (37). Besides, JAK1-STAT3 pathway up-regulated the expression of SOX9 and induced CEACAM5 overexpression, thus promoting breast cancer cell invasion (38). Meanwhile, there are research reporting CEACAM5 was major target genes for Smad3-mediated TGF-β signaling (39). Furthermore, the ACE2+SOX9+ double positive cells are readily infected by SARS-CoV-2 pseudovirus and significantly decreased in older children (40). These findings indicate that abnormal SOX9 could reduce CEACAM5 expression after SARS-CoV-2 spike stimulation, which may associate with JAK1-STAT3 pathway or Smad3-mediated TGF-β signaling. However, it requires further validation.

Apart from the elevation of inflammatory factors, cytokine storm was also manifested by severe CD4+ and CD8+ T cell lymphopenia and coagulopathy (41), which have been proposed as biomarkers for COVID severity (42–45). In addition to the lymphopenia induced by apoptosis in SARS-CoV-2 (46–49), researchers also observed the increased expression of T cell exhaustion markers, such as programmed cell death protein-1 (PD-1) and TIM-3 in peripheral blood of severe COVID-19 patients (44, 50). Except for the reduction in T cell numbers, researchers also found an increased frequency of activated T cell phenotypes (51). Furthermore, previous study has confirmed increased proportion of activated biomarkers HLA-DR and CD38 accompanied with reduced CD4+ and CD8+ T count (52). Although it has been confirmed that T lymphocytes in blood could be infected by SARS-CoV-2 in an ACE22/TMPRSS2-independent manner and the infection of T cells is likely to induce cell apoptosis in mitochondria ROS-HIF-1a-dependent pathways (47), little is known about the imbalance in T cell homeostasis and its mechanisms in intestinal barriers loss during SARS-CoV-2 infection. Our study found that immune abnormalities in GI after spike stimulation was characterized by the polarization of CD4+ T cells towards pro-inflammatory and increased apoptosis through inhibition of PI3K/AKT/mTOR pathway. Researchers also reported rapamycin and its analogs (rapalogs, including everolimus, temsirolimus, and ridaforolimus), as FDA-approved mTOR inhibitors, increased the susceptibility to SARS-CoV-2 infection in tissue culture and immunologically naive rodents (53). Taken together, maintenance of T cell homeostasis is crucial in the treatment of COVID-19 patients especially with GI symptoms.

Galectin-9, as a ligand to TIM-3, is expressed on several immune cells including T cells. It has been detected in the plasma of patients with viral infections such as HIV, influenza virus, hepatitis C virus (HCV), herpes simplex virus (HSV), human cytomegalovirus (HCMV), chronic hepatitis B virus (HBV) and dengue virus (DENV), indicating its important role in viral infection and pathogenesis (54). Besides, the expression of Galectin-9 was found to be significantly elevated in severe COVID-19 patients compared to convalescent patients and healthy individuals (50). It is found that plasma Galectin-9 has positive correlation with elevated proinflammatory cytokines and chemokines in COVID-19 patients. Researchers have further confirmed the overexpression of proinflammatory molecules in immune cells from COVID-19 patients once treated with Galectin-9 *in vitro* experiments (55). In our study, we observed CD4+ T lymphopenia and increased cytokines in intestine tissues of mice stimulated by SARS-CoV-2 spike. We also further revealed that the increased expression of Galectin-9 in CD4+ T cells could promote inflammatory factor release and increased apoptosis of CD4+ T. However, the specific mechanism of this interaction would worth further investigations, which might provide new insights and potential therapeutic targets for the treatment of cytokine storm in severe COVID patients.

In summary, our results demonstrated for the first time that the low expression of CEACAM5 upon SARS-CoV-2 spike stimulation induced intestinal barrier dysfunction through the interaction between CEACAM5 and Galectin-9. Increased expression of Galectin-9 promoted the polarization of CD4+ T cells towards pro-inflammatory phenotype. Then it elevated production of proinflammatory cytokines, inhibited PI3K/AKT/mTOR pathway and increased apoptosis of CD4+ T cells, eventually resulting in intestinal barrier dysfunction. Overexpression of CEACAM5 and knockdown of Galectin-9 displayed important role in maintaining intestinal barrier integrity. Based on these findings, targeting CEACAM5 and Galectin-9 could provide novel therapeutic strategies in intestinal barrier dysfunction of severe COVID patients and potential underlying mechanism remains to be further explored.

## Data availability statement

The original contributions presented in the study are included in the article/**Supplementary Materials**, further inquiries can be directed to the corresponding author/s.

## Ethics statement

The studies involving humans were approved by Medical Ethical Committee of the Fifth Affiliated Hospital of Sun Yat-sen University. The studies were conducted in accordance with the local legislation and institutional requirements. The participants provided their written informed consent to participate in this study. The animal study was approved by Experimental Animal Ethics Committee of the Fifth Affiliated Hospital, Sun Yat-sen University. The study was conducted in accordance with the local legislation and institutional requirements.

## Author contributions

YL: Writing – original draft, Conceptualization, Formal analysis, Investigation, Methodology, Project administration, Validation, Visualization, Writing – review & editing. ZZ: Writing – original draft, Conceptualization, Data curation, Formal analysis, Investigation. JR: Writing – original draft, Conceptualization, Data curation, Investigation, Methodology, Project administration, Supervision. CD: Data curation, Writing – review & editing, Methodology, Project administration. JW: Data curation, Writing – original draft, Supervision, Validation. YY: Data curation, Writing – original draft, Methodology, Supervision, Validation. XL: Conceptualization, Funding acquisition, Writing – original draft, Supervision, Writing – review & editing. ZY: Formal analysis, Software, Supervision, Writing – review & editing, Conceptualization, Methodology. YH: Conceptualization, Supervision, Writing – review & editing, Project administration, Validation.

## References

[1] Rader B Scarpino SV Nande A Hill AL Adlam B Reiner RC. Crowding and the shape of COVID-19 epidemics. Nat Med. (2020) 26:1829–34. doi: 10.1038/s41591-020-1104-0 33020651

[2] Huang C Wang Y Li X Ren L Zhao J Hu Y. Clinical features of patients infected with 2019 novel coronavirus in Wuhan, China. Lancet. (2020) 395:497–506. doi: 10.1016/S0140-6736(20)30183-5 31986264 PMC7159299

[3] Chen N Zhou M Dong X Qu J Gong F Han Y. Epidemiological and clinical characteristics of 99 cases of 2019 novel coronavirus pneumonia in Wuhan, China: a descriptive study. Lancet. (2020) 395:507–13. doi: 10.1016/S0140-6736(20)30211-7 PMC7135076 32007143

[4] Lin L Jiang X Zhang Z Huang S Zhang Z Fang Z. Gastrointestinal symptoms of 95 cases with SARS-CoV-2 infection. Gut. (2020) 69:997–1001. doi: 10.1136/gutjnl-2020-321013 32241899

[5] Xiao F Tang M Zheng X Liu Y Li X Shan H. Evidence for gastrointestinal infection of SARS-coV-2. Gastroenterology. (2020) 158:1831–3 e3. doi: 10.1053/j.gastro.2020.02.055 32142773 PMC7130181

[6] Jin X Lian JS Hu JH Gao J Zheng L Zhang YM. Epidemiological, clinical and virological characteristics of 74 cases of coronavirus-infected disease 2019 (COVID-19) with gastrointestinal symptoms. Gut. (2020) 69:1002–9. doi: 10.1136/gutjnl-2020-320926 PMC7133387 32213556

[7] Wan Y Li J Shen L Zou Y Hou L Zhu L. Enteric involvement in hospitalised patients with COVID-19 outside Wuhan. Lancet Gastroenterol Hepatol. (2020) 5:534–5. doi: 10.1016/S2468-1253(20)30118-7 PMC7159861 32304638

[8] Bestle D Heindl MR Limburg H Van Lam van T Pilgram O Moulton H. TMPRSS2 and furin are both essential for proteolytic activation of SARS-CoV-2 in human airway cells. Life Sci Alliance. (2020) 3:e202000786. doi: 10.1101/2020.04.15.042085 32703818 PMC7383062

[9] Hoffmann M Kleine-Weber H Schroeder S Kruger N Herrler T Erichsen S. SARS-coV-2 cell entry depends on ACE2 and TMPRSS2 and is blocked by a clinically proven protease inhibitor. Cell. (2020) 181:271–80 e8. doi: 10.1016/j.cell.2020.02.052 32142651 PMC7102627

[10] Hamming I Timens W Bulthuis ML Lely AT Navis G van Goor H. Tissue distribution of ACE2 protein, the functional receptor for SARS coronavirus. A first step in understanding SARS pathogenesis. J Pathol. (2004) 203:631–7. doi: 10.1002/path.1570 PMC7167720 15141377

[11] Kuba K Imai Y Rao S Gao H Guo F Guan B. A crucial role of angiotensin converting enzyme 2 (ACE2) in SARS coronavirus-induced lung injury. Nat Med. (2005) 11:875–9. doi: 10.1038/nm1267 PMC7095783 16007097

[12] Zeng FM Li YW Deng ZH He JZ Li W Wang L. SARS-CoV-2 spike spurs intestinal inflammation via VEGF production in enterocytes. EMBO Mol Med. (2022) 14:e14844. doi: 10.15252/emmm.202114844 35362189 PMC9081906

[13] Saia RS Giusti H Luis-Silva F Pedroso KJB Auxiliadora-Martins M Morejon KML. Clinical investigation of intestinal fatty acid-binding protein (I-FABP) as a biomarker of SARS-CoV-2 infection. Int J Infect Dis. (2021) 113:82–6. doi: 10.1016/j.ijid.2021.09.051 PMC8479553 34597762

[14] Assimakopoulos SF Mastronikolis S Al Del Aretha D Papageorgiou D Chalkidi T. Intestinal barrier biomarker ZO1 and endotoxin are increased in blood of patients with COVID-19-associated pneumonia. In Vivo. (2021) 35:2483–8. doi: 10.21873/invivo.12528 PMC8286488 34182534

[15] Ren Z Wang H Cui G Lu H Wang L Luo H. Alterations in the human oral and gut microbiomes and lipidomics in COVID-19. Gut. (2021) 70:1253–65. doi: 10.1136/gutjnl-2020-323826 PMC8042598 33789966

[16] Zuo T Zhang F Lui GCY Yeoh YK Li AYL Zhan H. Alterations in gut microbiota of patients with COVID-19 during time of hospitalization. Gastroenterology. (2020) 159:944–55 e8. doi: 10.1053/j.gastro.2020.05.048 32442562 PMC7237927

[17] He F Zhang T Xue K Fang Z Jiang G Huang S. Fecal multi-omics analysis reveals diverse molecular alterations of gut ecosystem in COVID-19 patients. Anal Chim Acta. (2021) 1180:338881. doi: 10.1016/j.aca.2021.338881 34538334 PMC8310733

[18] Bai X Wei H Liu W Coker OO Gou H Liu C. Cigarette smoke promotes colorectal cancer through modulation of gut microbiota and related metabolites. Gut. (2022) 71:2439–50. doi: 10.1136/gutjnl-2021-325021 PMC9664112 35387878

[19] Yang J Wei H Zhou Y Szeto CH Li C Lin Y. High-fat diet promotes colorectal tumorigenesis through modulating gut microbiota and metabolites. Gastroenterology. (2022) 162:135–49 e2. doi: 10.1053/j.gastro.2021.08.041 34461052

[20] Gold P Freedman SO. Specific carcinoembryonic antigens of the human digestive system. J Exp Med. (1965) 122:467–81. doi: 10.1084/jem.122.3.467 PMC2138078 4953873

[21] Toy LS Yio XY Lin A Honig S Mayer L. Defective expression of gp180, a novel CD8 ligand on intestinal epithelial cells, in inflammatory bowel disease. J Clin Invest. (1997) 100:2062–71. doi: 10.1172/JCI119739 PMC508397 9329971

[22] Roda G Jianyu X Park MS DeMarte L Hovhannisyan Z Couri R. Characterizing CEACAM5 interaction with CD8alpha and CD1d in intestinal homeostasis. Mucosal Immunol. (2014) 7:615–24. doi: 10.1038/mi.2013.80 PMC3981948 24104458

[23] Allez M. A CEACAM5-derived peptide activating CD8(+) regulatory T cells: A future option for restoring mucosal homeostasis in crohn’s disease? Gastroenterology. (2022) 163:822–4. doi: 10.1053/j.gastro.2022.07.070 35931106

[24] Gu S Zaidi S Hassan MI Mohammad T Malta TM Noushmehr H. Mutated CEACAMs disrupt transforming growth factor beta signaling and alter the intestinal microbiome to promote colorectal carcinogenesis. Gastroenterology. (2020) 158:238–52. doi: 10.1053/j.gastro.2019.09.023 PMC7124154 31585122

[25] Alhetheel A Albarrag A Shakoor Z Somily A Barry M Altalhi H. Differential expression of carcinoembryonic antigen-related cell adhesion molecule-5 (CEACAM5) and dipeptidyl peptidase-4 (DPP4) with detection of Middle East respiratory syndrome-coronavirus in peripheral blood. J Infect Public Health. (2022) 15:1315–20. doi: 10.1016/j.jiph.2022.10.008 PMC9576204 36279687

[26] Mazgaeen L Yorek M Saini S Vogel P Meyerholz DK Kanneganti TD. CD47 halts Ptpn6-deficient neutrophils from provoking lethal inflammation. Sci Adv. (2023) 9:eade3942. doi: 10.1126/sciadv.ade3942 36608128 PMC9821860

[27] Zhou J Li C Liu X Chiu MC Zhao X Wang D. Infection of bat and human intestinal organoids by SARS-CoV-2. Nat Med. (2020) 26:1077–83. doi: 10.1038/s41591-020-0912-6 32405028

[28] Lamers MM Beumer J van der Vaart J Knoops K Puschhof J Breugem TI. SARS-CoV-2 productively infects human gut enterocytes. Science. (2020) 369:50–4. doi: 10.1126/science.abc1669 PMC7199907 32358202

[29] Deng W Bao L Liu J Xiao C Liu J Xue J. Primary exposure to SARS-CoV-2 protects against reinfection in rhesus macaques. Science. (2020) 369:818–23. doi: 10.1126/science.abc5343 PMC7402625 32616673

[30] Chandrashekar A Liu J Martinot AJ McMahan K Mercado NB Peter L. SARS-CoV-2 infection protects against rechallenge in rhesus macaques. Science. (2020) 369:812–7. doi: 10.1126/science.abc477 PMC7243369 32434946

[31] Sia SF Yan LM Chin AWH Fung K Choy KT Wong AYL. Pathogenesis and transmission of SARS-CoV-2 in golden hamsters. Nature. (2020) 583:834–8. doi: 10.1038/s41586-020-2342-5 PMC7394720 32408338

[32] Imai M Iwatsuki-Horimoto K Hatta M Loeber S Halfmann PJ Nakajima N. Syrian hamsters as a small animal model for SARS-CoV-2 infection and countermeasure development. Proc Natl Acad Sci U S A. (2020) 117:16587–95. doi: 10.1073/pnas.2009799117 PMC7368255 32571934

[33] Giron LB Dweep H Yin X Wang H Damra M Goldman AR. Plasma markers of disrupted gut permeability in severe COVID-19 patients. Front Immunol. (2021) 12:686240. doi: 10.3389/fimmu.2021.779064 34177935 PMC8219958

[34] Peng X Ouyang J Isnard S Lin J Fombuena B Zhu B. Sharing CD4+ T cell loss: when COVID-19 and HIV collide on immune system. Front Immunol. (2020) 11:596631. doi: 10.3389/fimmu.2020.596631 33384690 PMC7770166

[35] Martonik D Parfieniuk-Kowerda A Rogalska M Flisiak R. The role of th17 response in COVID-19. Cells. (2021) 10:1550. doi: 10.3390/cells10061550 34205262 PMC8235311

[36] Jiao L Li H Xu J Yang M Ma C Li J. The gastrointestinal tract is an alternative route for SARS-coV-2 infection in a nonhuman primate model. Gastroenterology. (2021) 160:1647–61. doi: 10.1053/j.gastro.2020.12.001 PMC7725054 33307034

[37] Jay P Berta P Blache P. Expression of the carcinoembryonic antigen gene is inhibited by SOX9 in human colon carcinoma cells. Cancer Res. (2005) 65:2193–8. doi: 10.1158/0008-5472.CAN-04-1484 15781631

[38] Yang H Geng YH Wang P Yang H Zhou YT Zhang HQ. Extracellular ATP promotes breast cancer invasion and chemoresistance via SOX9 signaling. Oncogene. (2020) 39:5795–810. doi: 10.1038/s41388-020-01402-z 32724162

[39] Han SU Kwak TH Her KH Cho YH Choi C Lee HJ. CEACAM5 and CEACAM6 are major target genes for Smad3-mediated TGF-beta signaling. Oncogene. (2008) 27:675–83. doi: 10.1038/sj.onc.1210686 17653079

[40] Zhang Z Guo L Lu X Zhang C Huang L Wang X. Clinical analysis and pluripotent stem cells-based model reveal possible impacts of ACE2 and lung progenitor cells on infants vulnerable to COVID-19. Theranostics. (2021) 11:2170–81. doi: 10.7150/thno.53136 PMC7797681 33500718

[41] Copaescu A Smibert O Gibson A Phillips EJ Trubiano JA. The role of IL-6 and other mediators in the cytokine storm associated with SARS-CoV-2 infection. J Allergy Clin Immunol. (2020) 146:518–34 e1. doi: 10.1016/j.jaci.2020.07.001 32896310 PMC7471766

[42] Wu C Chen X Cai Y Xia J Zhou X Xu S. Risk factors associated with acute respiratory distress syndrome and death in patients with coronavirus disease 2019 pneumonia in Wuhan, China. JAMA Intern Med. (2020) 180:934–43. doi: 10.1001/jamainternmed.2020.0994 PMC7070509 32167524

[43] Chen G Wu D Guo W Cao Y Huang D Wang H. Clinical and immunological features of severe and moderate coronavirus disease 2019. J Clin Invest. (2020) 130:2620–9. doi: 10.1172/JCI137244 PMC7190990 32217835

[44] Diao B Wang C Tan Y Chen X Liu Y Ning L. Reduction and functional exhaustion of T cells in patients with coronavirus disease 2019 (COVID-19). Front Immunol. (2020) 11:827. doi: 10.3389/fimmu.2020.00827 32425950 PMC7205903

[45] Zheng M Gao Y Wang G Song G Liu S Sun D. Functional exhaustion of antiviral lymphocytes in COVID-19 patients. Cell Mol Immunol. (2020) 17:533–5. doi: 10.1038/s41423-020-0402-2 PMC7091858 32203188

[46] Xiong Y Liu Y Cao L Wang D Guo M Jiang A. Transcriptomic characteristics of bronchoalveolar lavage fluid and peripheral blood mononuclear cells in COVID-19 patients. Emerg Microbes Infect. (2020) 9:761–70. doi: 10.1080/22221751.2020.1747363 PMC7170362 32228226

[47] Shen XR Geng R Li Q Chen Y Li SF Wang Q. ACE2-independent infection of T lymphocytes by SARS-CoV-2. Signal Transduct Target Ther. (2022) 7:83. doi: 10.1038/s41392-022-00919-x 35277473 PMC8914143

[48] Andre S Picard M Cezar R Roux-Dalvai F Alleaume-Butaux A Soundaramourty C. T cell apoptosis characterizes severe Covid-19 disease. Cell Death Differ. (2022) 29:1486–99. doi: 10.1038/s41418-022-00936-x PMC8782710 35066575

[49] Adamo S Chevrier S Cervia C Zurbuchen Y Raeber ME Yang L. Profound dysregulation of T cell homeostasis and function in patients with severe COVID-19. Allergy. (2021) 76:2866–81. doi: 10.1111/all.14866 PMC8251365 33884644

[50] Schultheiss C Paschold L Simnica D Mohme M Willscher E von Wenserski L. Next-generation sequencing of T and B cell receptor repertoires from COVID-19 patients showed signatures associated with severity of disease. Immunity. (2020) 53:442–55 e4. doi: 10.1016/j.immuni.2020.06.024 32668194 PMC7324317

[51] Mathew D Giles JR Baxter AE Oldridge DA Greenplate AR Wu JE. Deep immune profiling of COVID-19 patients reveals distinct immunotypes with therapeutic implications. Science. (2020) 369:eabc8511. doi: 10.1126/science.abc8511 32669297 PMC7402624

[52] Xu Z Shi L Wang Y Zhang J Huang L Zhang C. Pathological findings of COVID-19 associated with acute respiratory distress syndrome. Lancet Respir Med. (2020) 8:420–2. doi: 10.1016/S2213-2600(20)30076-X PMC7164771 32085846

[53] Shi G Chiramel AI Li T Lai KK Kenney AD Zani A. Rapalogs downmodulate intrinsic immunity and promote cell entry of SARS-CoV-2. J Clin Invest. (2022) 132:e160766. doi: 10.1172/JCI160766 36264642 PMC9753997

[54] Merani S Chen W Elahi S. The bitter side of sweet: the role of Galectin-9 in immunopathogenesis of viral infections. Rev Med Virol. (2015) 25:175–86. doi: 10.1002/rmv.1832 25760439

[55] Bozorgmehr N Mashhouri S Perez Rosero E Xu L Shahbaz S Sligl W. Galectin-9, a player in cytokine release syndrome and a surrogate diagnostic biomarker in SARS-coV-2 infection. mBio. (2021) 12:e00384-21. doi: 10.1128/mBio.00384-21 33947753 PMC8262904

