# Supplementary material for: SARS-Cov-2 spike induces intestinal barrier dysfunction through the interaction between CEACAM5 and Galectin-9
Source: Front Immunol. 2024 Apr 15;15:1303356. doi: 10.3389/fimmu.2024.1303356 (PMC11056506; doi:10.3389/fimmu.2024.1303356)
Supplement: Supplementary file 1 [file Table_1.docx]

**Supplemental Table 1.** The sequences of the primers used in this assay

| Name | Forward primer | Reverse primer |
| --- | --- | --- |
| Homo-IL-4 | AACTTTGAACAGCCTCACAGAGC | TTCTCATGGTGGCTGTAGAACTG |
| Homo-IL-10 | CCTGCCTAACATGCTTCGAGAT | GGCAACCCAGGTAACCCTTAAA |
| Homo-IL-17 | ATGACTCCTGGGAAGACCTCATT | TCCGGTTATGGATGTTCAGGTTG |
| Homo-IFN-γ | CCAAAAGAGTGTGGAGACCATCA | CATGTATTGCTTTGCGTTGGACA |
| Homo-GAPHD | ACAGCCTCAAGATCATCAGCA | ATGAGTCCTTCCACGATACCA |
| Mus- GAPHD | GGTCCCAGCTTAGGTTCATCA | CCAATACGGCCAAATCCGTTC |
| Mus-IL-4 | ATCATCGGCATTTTGAACGAGGTC | ACCTTGGAAGCCCTACAGACGA |
| Mus- IL-10 | CGGGAAGACAATAACTGCACCC | CGGTTAGCAGTATGTTGTCCAGC |
| Mus- IL-17 | CAGACTACCTCAACCGTTCCAC | TCCAGCTTTCCCTCCGCATTGA |
| Mus- IFN-γ | CAGCAACAGCAAGGCGAAAAAGG | TTTCCGCTTCCTGAGGCTGGAT |
